# 

**Published:** 1893-11

**Authors:** 


### NOTICE.

### The Ohio State Dental Society.

The present prospects for an extra good meeting of this society are flattering. The Executive Committee have been faithfully working in the interests of the society and the program will be completed in a few days. Arrangements have been made to hold all meetings and clinics at the Neil House, Columbus; a large, well-lighted room has been secured specially for clinics and exhibits.

The following is but a portion of the program to be presented, but as much as can be given at this writing, on account of several delayed responses from members who are to write papers or clinic.

ESSAYS.

President's Address, DR. G. H. WILSON, Cleveland.

The Bonwill Method of Articulation,
DR. GRANT MOLYNEAUX, Cincinnati.

Sulphuric Acid for Opening Root Canals,
DR. J. R. CALLAHAN, Cincinnati.

Inflammation, - - Dr. J. Taft, Cincinnati.
A Serious Paper, - Dr. C. M. Wright, Cincinnati.
"Vis Medicatrix Naturæ," Dr. W. H. Whitslar, Cleveland.
——————— Dr. L. E. Custer, Dayton.
Congenital Deformities of the Superior Maxillary,
Prof T. C. Hoover, M.D., Columbus.
Therapeutics of Tr. Iodine and Aconite in Pericementitis,
W. I. Jones, Columbus.
Report of the State Board of Dental Examiners,
Dr. Grant Mitchell, Canton.

CLINICS.

Bonwill Method of Articulation,
Dr. Grant Molyneaux, Cincinnati.
Demonstration of The Hollingsworth System for Crowns and Bridges, - Dr. G. F. Houser, Kansas City, Mo.
Making and Setting Crowns made in Downey Furnace,
Dr. L. L. Barber, Toledo.
A Clinical Lecture, - Dr. J. Taft, Cincinnati.
Porcelain Inlay, etc., G. Mitchell, Canton.
Exhibition and Demonstration of the Custer Electrical Cabinet,
Dr. L. E. Custer, Dayton.

Arrangements have been completed for cheap railroad transportation of one and one-third fare to all who secure a certificate of the agent when purchasing ticket to Columbus.

No Dentist in Ohio can afford to miss this meeting, as the clinics and papers will be of special value. Cross Tuesday, Wednesday, Thursday and Friday, December, 5th, 6th, 7th and 8th, 1893, off your appointment book, and assist in making this the largest and best meeting ever held.

### EDITORIAL.

### Ohio State Dental Society.

This body will hold its annual meeting at the Neil House, in Columbus, December 5th to 8th inclusive, 1893.

This will be an important meeting of this society, and every member should be present; and it is hoped that many members of the profession, who are not yet members of this Society, will be present and identify themselves with it. This is one of the oldest State Dental Societies, and it is on this account of interest to every progressive dentist in the State. But more than this it should be appreciated for what it has done for the profession: Through it dental legislation was obtained for regulating the practice of dentistry in the State, and because of the success attained in this direction many other States were induced to do likewise. Perhaps, in no other State in the Union is the profession in a better condition than in Ohio, and due mainly to the work done by this society.

Let every member see to it that he is present and prepared to do something for his fellows; and not only this, but let every member so far as possible use his best efforts to bring one or more dentists who are not as yet members. The indications are that this will be a large and interesting meeting.

The influence of The World's Dental Congress was somewhat adverse to good meetings of the various State Societies for the last two years, but now that is past, and the resources should be gathered and utilized to the best advantage.

Everybody come and bring his neighbor.

### The Dental Register,

A Monthly Magazine Devoted to the Interests of the

### DENTAL PROFESSION.

*Terms Reduced to* $2.00 *Per Year. Single Copies,* 25 *Cents.*

### EDITED BY PROF. J. TAFT, D.D.S.

——AND——

### Published by SAM'L A. CROCKER & CO., Ohio Dental and Surgical Depot.

The volume commences in January and closes in December.

The Register being issued monthly is always fresh, therefore Indispensable to the practitioner who desires to keep posted up with the new inventions and improvements which are daily developed. Neither expense nor effort will be spared to give value and variety to its contents Articles will be illustrated with well executed engravings whenever they will add to the interest or assist to explanation.

Its extensive circulation and frequency of issue make it one of the BEST DENTAL ADVERTISING MEDIUMS in the United States.

All subscriptions must begin with January or July.

Local or special notices, five lines or less, will be inserted for $3 each insertion; over five lines, 50 cts. per line.

All advertising bills payable in advance, or at furthest, after the issue of the first number containing the advertisement. All transient advertisements to be paid for in advance.

☞CONTRIBUTIONS SOLICITED.

Exclusively Editorial Communications should be addressed to the Editor.

The latest number of the Register may be ordered with or without other good' at any time, and all letters should be addressed to

